# Knowledge and Practice of Personal Hygiene Among the Primary School-Going Children in a Rural District of Assam, India

**DOI:** 10.7759/cureus.90694

**Published:** 2025-08-21

**Authors:** Himakshi Devi, Rajib Ray Baruah, Narayan C Sharma, Shankhadhwaj Borah, Putul Mahanta

**Affiliations:** 1 Nursing, Hayat Institute of Nursing Education, Srimanta Sankaradeva University of Health Sciences (SSUHS), Guwahati, IND; 2 Pediatric Surgery, Gauhati Medical College and Hospital, Guwahati, IND; 3 Pediatrics, PA Sangma International Medical College and Hospital, Khanapara, IND; 4 Otolaryngology, Sonari District Hospital, Sonari, IND; 5 Forensic Medicine and Toxicology, Nalbari Medical College (NMC), Nalbari, IND

**Keywords:** behavior, health, knowledge, personal hygiene, practice

## Abstract

Introduction

Early education in proper hygiene practices is crucial for stopping the spread of infectious diseases in children. The present study aims to assess the knowledge and practices of personal hygiene among the primary school-going children in a rural area of northeast India.

Methods

This cross-sectional, descriptive, quantitative study was conducted among 120 primary school-going children from seven government schools in the Kamrup (Rural) District, Assam. A structured interview schedule was used to collect data on the knowledge and practices of personal hygiene among the participants. An observation checklist was used to assess the personal hygiene practices of primary school children.

Results

Parents (n = 88; 73.3%) and teachers (n = 17; 14.2%) were the primary sources of information on personal hygiene. A majority of 70 (58.30%) participants had moderately adequate knowledge, and 84 (70%) exhibited average practices of personal hygiene, with a significant positive correlation (r = 0.68; p < 0.001) between the knowledge and practice scores. Parents' occupations (p-value = 0.001), sources of information (p-value = 0.01), and religion (p-value < 0.001) were associated with personal hygiene practices.

Conclusion

The majority of participants were aware of and exercised personal hygiene to a moderate extent. Although knowledge and personal hygiene practices were positively correlated, a significant discrepancy existed between the children's stated knowledge and practice levels and the observed personal hygiene indicators.

## Introduction

The practice of keeping one's external body clean and well-groomed is known as personal hygiene. Maintaining personal hygiene is important for social health, psychological well-being, and simply as a way of life. Encouraging children's good health boosts the growth of the family, nation, and planet. The health status is considered an index of a nation's development. Personal hygiene is crucial for maintaining good health [1]. Children can be taught good hygiene habits from a young age, which are essential to preventing the spread of infectious diseases. Washing hands, brushing teeth, taking a shower, and wearing clean clothes are all part of maintaining proper personal hygiene. It's also about making hygienic and safe choices. In addition to societal, familial, and personal factors, an individual's knowledge and attitudes on hygiene also have a significant impact on hygienic behaviors [2].

In India, diarrhea accounts for 13% of all deaths in children under the age of five each year, making it the third most common cause of childhood mortality [3]. In India, the high rate of morbidity and mortality among children from acute respiratory infections (ARIs) is a serious public health concern [4]. Handwashing with soap and water has been shown to lower respiratory and diarrheal illnesses by 17% and 30%, respectively, in low- and middle-income countries [5,6]. Untreated caries in deciduous teeth affects more than half a billion children globally, potentially having a major negative influence on their quality of life. Dental caries is one of the most common multifactorial, preventable diseases, which can be largely prevented through nutrition and oral hygiene [7]. A recent study also documented that soil-transmitted helminthic (STH) infection was more likely to occur in children who had irregular handwashing before meals and exhibited poor handwashing behavior in school [8]. India has a high prevalence of STH infections, especially in preschool and school-aged children. STH can be effectively controlled and prevented through mass drug administration and enhanced water, sanitation, and hygiene practices [9]. The WHO/UNICEF Joint Monitoring Program for Water Supply, Sanitation, and Hygiene 2023 update estimated that in 2022, 27% of the world's population did not have access to "safely managed drinking water," 43% of the world's population lacked "safely managed sanitation," and 25% of the world's population lacked access to a handwashing station with soap and water at home [10]. Social rejection can also result from inadequate hygiene, particularly for children from lower-income households [11].

Parents, caregivers, and peers can influence the way children approach personal hygiene. Children are more likely to suffer from illnesses linked to poor personal hygiene since they are still learning how to take care of themselves and are exposed to a high number of germs in play areas and schools. Providing easy access to personal hygiene and health education is a straightforward and cost-effective approach for preventing and managing hygiene-related health concerns. The Indian government's "Swachh Bharat Swachh Vidyalaya" campaign was launched in 2014 to encourage schoolchildren to adopt safe and hygienic practices. The present study aims to assess the knowledge and practices of personal hygiene among the primary school-going children in a rural area of northeast India.

## Materials and methods

A cross-sectional, descriptive, quantitative study was conducted among primary school-going children in the 3rd, 4th, and 5th standards of seven government schools in Kamalpur Block, Kamrup (Rural) District, Assam. A total of 120 participants were included in the study. The participants were selected using a multistage random sampling technique. Out of the total 100 government primary schools in the study block, seven schools were selected by simple random sampling techniques. From each school, the number of participants was selected using the proportionate sampling technique. The students were picked up equally from each standard using the systematic random sampling technique. Ethical approval was obtained from the Institutional Ethical Committee of the Regional College of Nursing, Guwahati, Assam. 

Prior permission was obtained from the school authorities of the selected schools for conducting the research. At least one parent of the students was requested to attend the school on the day of data collection. The class teachers were informed about the purpose and nature of the study. The parents were also informed about the purpose of the study and assured that their children's information would be kept strictly confidential. Informed consent was obtained from one of the parents of the students who were willing to participate in the study. Also, verbal consent was obtained from the students to participate in the study.

Study setting

The present study included primary school children from seven government primary schools in Kamalpur Block, Kamrup (Rural) District, Assam. The study population comprised all primary school-going children in 3rd, 4th, and 5th standards studying at Laukuri L.P. School, Kacharua Jayram L.P. School, Kacharua Moktab L.P. School, Hahara L.P. School, 3 No. Bamungaon L.P. School, Mohan Kalita L.P. School, and Dekarkuchi L.P. School.

Sample size determination

Sample size was calculated using the formula: n = (z2P(1 - P))/d2, where Z is the value from the standard normal distribution corresponding to the desired confidence level, P is the expected true proportion, and d is the desired precision

The knowledge levels of primary school children varied across various studies conducted in India. A study from Coimbatore reported that only 66.7% of students demonstrated good knowledge of personal hygiene [12]. A similar study from Uttarakhand, India, observed that 83% of primary school children had fair knowledge regarding personal hygiene [13]. Considering a p-value of 83%, the sample size was determined to be 111 with a 95% confidence level and 7% precision. The final sample size was rounded off to 120.

Sample selection

A multistage random sampling technique was employed in the present study. At the first stage, seven schools were selected by the simple random sampling technique. Based on the total number of students enrolled in 3rd, 4th, and 5th standards of the school, the total number of participants from each school was determined using a proportionate sampling technique at stage 2. Then, the students were picked up equally from each standard (3rd, 4th, and 5th) using the systematic random sampling technique. A total of 120 students were thus selected for the study (Table 1).

**Table 1:** Number of students proportionately selected from each school LPS: lower primary school

Name of selected schools	Total number of students in 3rd, 4th, and 5th standards	Selected number of students from the school
Laukuri LPS	34	25
Kacharua Jayram LPS	15	11
Kacharua Moktab LPS	16	12
Hahara LPS	18	13
3 No Bamungaon LPS	33	24
Mohan Kalita LPS	30	22
Dekarkuchi LPS	18	13
Total	164	120

Inclusion and exclusion criteria

Children in the 3rd, 4th, and 5th standards who were willing to give consent to participate in the study and were present at the time of data collection were included. Children who were acutely ill during data collection and those who did not give consent to participate in the study were excluded.

Data collection tool

A structured interview schedule was used to collect the data. The first part of the tool consisted of demographic details about the participants, including age, gender, religion, type of family, standards, sources of information, father's occupation, and mother's occupation. 

The second part of the questionnaire assessed the knowledge of personal hygiene among primary school children and included 12 items with yes and no options. The positive response received a score of one, and the negative response received a score of zero. The total score was 12. The scores were divided into three categories: inadequate, moderately adequate, and adequate, based on the mean and standard deviation of knowledge scores. The third part included 12 items with yes/no options related to practices. The positive response scored one, and the negative response scored zero, with a total score of twelve. The scores were divided into three categories: poor, average, and good, based on the mean and standard deviation of practice scores. Finally, the fourth part of the tool included an observation checklist comprising ten items to identify personal hygiene practices among primary school children.

The tool was given to seven experts from various medical fields, and they were asked to provide their opinions and rate the item for relevance, accuracy, and appropriateness. Modifications were made according to the expert's suggestion. A pilot study involving 21 primary school children was conducted at two schools within the study block. The reliability of the tool was assessed using the Spearman-Brown coefficient split-half method and was found to be 0.80 for knowledge items and 0.81 for practice items, indicating a level of reliability that is fairly reliable and adequate for the study.

Statistical analysis

The data were analysed using IBM SPSS Statistics for Windows, Version 21 (Released 2012; IBM Corp., Armonk, New York, United States). The mean and standard deviation were used to present the continuous variables. At the same time, percentages and frequencies were used to display categorical variables. The Pearson correlation coefficient was used to assess the strength of association between knowledge and practice scores. The chi-square test was used to examine the association between knowledge and practice scores and sociodemographic variables. The p-value below 0.05 was deemed statistically significant.

## Results

Sociodemographic characteristics of the participants

Out of 120 primary school children included in the study, the majority, 55 (45.8%), belonged to the 9- to 10-year age group. Half of the participants were female (n = 66, 55%) and Hindu (n = 66, 55%). Participants primarily came from nuclear families (n = 103, 85.8%). The father's occupation was mostly cultivation (n = 63; 52.5%) and business (n = 26; 21.7%). At the same time, 81.7% (n = 98) of the mothers were homemakers. Most participants received information on personal hygiene from their parents (n = 88; 73.3%) and teachers (n = 17; 14.2%) (Table 2).

**Table 2:** Sociodemographic distribution of the participants The data are represented as frequency (n) and percentage (%), with a total sample size of N = 120

Variables	Categories	Frequency (f)	Percentage (%)
Age (in years)	7-8 years	14	11.7
	8-9 years	30	25.0
	9-10 years	55	45.8
	>10 years	21	17.5
Sex	Male	54	45.0
	Female	66	55.0
Religion	Hindu	66	55.0
	Muslim	54	45.0
Family type	Nuclear	103	85.8
	Joint	17	14.2
Occupation of the father	Govt. service	6	5.0
	Private service	22	18.3
	Business	26	21.7
	Farmer	63	52.5
	Unemployed	3	2.5
Occupation of the mother	Govt. service	3	2.5
	Private service	5	4.1
	Business	14	11.7
	Housewife	98	81.7
Source of information	Parents	88	73.3
	Teacher	17	14.2
	Siblings	8	6.7
	Books	7	5.8

Knowledge of personal hygiene among the participants

Most participants demonstrated good knowledge regarding regular bathing (n = 119, 99.2%), the methods of maintaining personal hygiene (n = 117, 97.5%), and its importance (n = 109, 90.8%). Only 64.2% (n = 77) of participants believed washing hands with soap was better than washing hands with water only. Above 80.0% of the participants responded positively that personal hygiene is important for good health (n = 106; 88.3%) and can prevent diseases (n = 103; 85.8%). Almost 89.2% (n = 107) of participants knew regular brushing, and 87.5% (n = 105) opined that nail biting is unhealthy. However, only 62.5% (n = 75) knew the disadvantages of eating too much sugar (Table 3).

**Table 3:** Knowledge regarding personal hygiene among the participants The data are represented as frequency (n) and percentage (%), with a total sample size of N = 120

Sl no.	Knowledge questions	Response Yes	
		Frequency	%
1	Personal hygiene means maintaining the body's cleanliness	104	86.7
2	Personal hygiene encompasses activities such as taking baths, washing hands, brushing teeth, using soap, trimming nails, and caring for gums	117	97.5
3	Maintaining personal hygiene is essential for everyone	109	90.8
4	Being neat and clean keeps you healthy	106	88.3
5	Taking a bath every day is necessary to keep you clean	119	99.2
6	Washing hands with soap is more effective than just using water	77	64.2
7	Maintaining personal hygiene can help prevent the contraction of diseases	103	85.8
8	Brushing your teeth regularly with toothpaste helps prevent tooth decay and other dental problems	107	89.2
9	Biting your nails with your teeth is a bad habit	105	87.5
10	Consuming excessive sugar can harm the teeth	75	62.5
11	Hair should be kept clean to prevent dandruff	97	80.8
12	You can gain knowledge about personal hygiene from your parents, TV, books, and other sources	97	80.8

Personal hygiene practices among the participants

All participants demonstrated good hand hygiene practices after defecating (n = 120; 100.0%) and before eating food (n = 118; 98.3%). The use of hand sanitiser at school was infrequent (n = 30, 25.0%). Almost 97% (n = 117) of participants reported brushing their teeth regularly before breakfast. But only 34.5% (n = 41) brushed their teeth after dinner, 59.7% (n = 71) brushed their teeth after eating sweets, and only 54.2% (n = 65) visited dentists. Almost 89.2% (n = 107) of participants practiced regular bathing, but only 47.5% (n = 57) washed their hair regularly (Table 4).

**Table 4:** Practice regarding personal hygiene among the participants The data are represented as frequency (n) and percentage (%), with a total sample size of N = 120

Sl no.	Practice questions	Response Yes	
		Frequency	%
1	Do you wash your hands with soap before eating?	118	98.3
2	Do you wash your hands with soap after using the toilet?	120	100.0
3	Do you wash your hands after playing?	89	74.2
4	Do you use soap while washing your hands?	101	84.2
5	Do you use hand sanitiser instead of hand washing in school?	30	25.0
6	Do you brush your teeth before breakfast?	117	97.5
7	Do you brush your teeth after dinner?	41	34.5
8	Do you rinse your teeth after eating sweets?	71	59.7
9	Do you visit the dentist yearly?	65	54.2
10	Do you take baths regularly?	107	89.2
11	Do you wash your hair daily?	57	47.5
12	Do your parents or family members help you in taking a bath?	114	95.0

Distribution of knowledge and practice score of personal hygiene and its association

The knowledge scores were categorized as inadequate if the knowledge score was <9, moderately adequate if the knowledge score was 9-11, and adequate if the knowledge score was >11. Similarly, to assess the practices of personal hygiene among primary school children, the practice scores were categorized as poor if the score was <7, average if the score was 7-10, and good if the score was >10.

As shown in Table 5, the majority of 70 participants (58.30%) had moderately adequate knowledge, and 32 participants (26.70%) had adequate knowledge of personal hygiene. Also, 84 (70%) had average practices, and 17 (14.20%) had good practices of personal hygiene. The correlation analysis revealed a significant positive correlation (r = 0.68; p < .001) between the knowledge and practice scores.

**Table 5:** Distribution of knowledge and practice scores of the participants The data are represented as mean, standard deviation (SD), frequency (n), and percentage (%); the total sample size is N = 120

Variables	Mean ± SD	Level of knowledge	Frequency (%)
Knowledge score	10.3 ± 1.5	Inadequate (<9)	18 (15.0%)
		Moderately adequate (9-11)	70 (58.3%)
		Adequate (>11)	32 (26.7%)
Practices score	8.6 ± 1.7	Poor (<7)	19 (15.8%)
		Average (7-10)	84 (70.0%)
		Good (>10)	17 (14.2%)

Association of knowledge and practice of personal hygiene with sociodemographic variables

As shown in Table 6, a significant association was observed between the knowledge score and religion (p-value < 0.001) and father's occupation (p-value = 0.001). However, no notable association was observed between knowledge scores and age (p-value = 0.13), gender (p-value = 0.62), type of family (p-value = 0.34), mother's occupation (p-value = 0.15), or source of information (p-value = 0.81).

**Table 6:** Association of knowledge score with sociodemographic variables The data are represented as frequency (n) and percentage (%), with a total sample size of N = 120. *p < 0.05 was considered statistically significant for the chi-square test

Variables	Categories	Knowledge score			χ^2^ value	p-value
		Inadequate (n = 18)	Moderately adequate (n = 70)	Adequate (n = 32)		
Age	7-8 years (n = 14)	3 (21.4%)	10 (71.4%)	1 (7.1%)	χ^2^ = 9.82	0.13
	8-9 years (n = 30)	7 (23.3%)	14 (46.7%)	9 (30.0%)		
	9-10 years (n = 55)	6 (10.9%)	30 (54.5%)	19 (34.5%)		
	>10 years (n = 21)	2 (9.5%)	16 (76.2%)	3 (14.3%)		
Gender	Male (n = 54)	10 (18.5%)	30 (55.6%)	14 (25.9%)	χ^2 ^= 0.96	0.62
	Female (n = 66)	8 (12.1%)	40 (60.6%)	18 (27.3%)		
Religion	Hindu (n = 66)	3 (4.5%)	36 (54.5%)	27 (40.9%)	χ^2 ^= 22.2	<0.001*
	Muslim (n = 54)	15 (27.8%)	34 (63.0%)	5 (9.2%)		
Type of family	Nuclear (n = 103)	16 (15.5%)	62 (60.2%)	25 (24.3%)	χ^2 ^= 2.13	0.34
	Joint (n = 17)	2 (1.9%)	8 (7.8%)	7 (6.8%)		
Father's occupation	Govt. job (n = 6)	0 (0.0%)	2 (33.3%)	4 (66.6%)	χ^2 ^= 26.31	0.001*
	Private job (n = 22)	0 (0.0%)	12 (54.5%)	10 (45.5%)		
	Business (n = 26)	0 (0.0%)	18 (69.2%)	8 (30.8%)		
	Farmer (n = 63)	17 (27.0%)	36 (57.1%)	10 (15.9%)		
	Unemployed (n = 3)	1 (33.3%)	2 (66.7%)	0 (0.0%)		
Mother's occupation	Govt. job (n = 3)	0 (0.0%)	0 (0.0%)	3 (100.0%)	χ^2 ^= 9.40	0.15
	Private job (n = 5)	1 (20.0%)	3 (60.0%)	1 (20.0%)		
	Business (n = 14)	1 (7.1%)	9 (64.3%)	4 (28.6%)		
	Housewife (n = 98)	16 (16.3%)	58 (59.2%)	24 (24.5%)		
Sources of information	Parents (n = 88)	15 (17.0%)	49 (55.7%)	24 (27.3%)	χ^2 ^= 2.95	0.81
	Siblings (n = 8)	1 (12.5%)	5 (62.5%)	2 (25.0%)		
	Teacher (n = 17)	2 (11.8%)	10 (58.8%)	5 (29.4%)		
	Books (n = 7)	0 (0.0%)	6 (85.7%)	1 (14.3%)		

Similarly, in the case of personal hygiene practices among the participants, the occupation of both parents (p-value = 0.001), sources of information (p-value = 0.01), and religion (p-value < 0.001) were significantly associated with the students' practice scores (Table 7).

**Table 7:** Association of practice score with sociodemographic variables The data are represented as frequency (n) and percentage (%), with a total sample size of N = 120. *p < 0.05 was considered statistically significant for the chi-square test

Variables	Categories	Practice score			χ^2^ value	p = value
		Poor (n = 19)	Average (n = 84)	Good (n = 17)		
Age	7-8 years (n = 14)	3 (21.4%)	8 (57.1%)	3 (21.4%)	χ^2^ = 2.04	0.91
	8-9 years (n = 30)	4 (13.3%)	23 (76.7%)	3 (1.0%)		
	9-10 years (n = 55)	8 (14.5%)	39 (70.9%)	8 (14.5%)		
	>10 years (n = 21)	4 (19.0%)	14 (66.7%)	3 (14.3%)		
Gender	Male (n = 54)	12 (22.2%)	34 (63.0%)	8 (14.8%)	χ^2 ^= 3.25	0.20
	Female (n = 66)	7 (10.6%)	50 (75.8%)	9 (13.6%)		
Religion	Hindu (n = 66)	3 (4.5%)	49 (74.2%)	14 (21.2%)	χ^2 ^= 17.3	<0.001*
	Muslim (n = 54)	16 (29.6%)	35 (64.8%)	3 (5.6%)		
Type of family	Nuclear (n = 103)	17 (16.5%)	73 (70.9%)	13 (12.6%)	χ^2 ^= 1.51	0.47
	Joint (n = 17)	2 (11.8%)	11 (64.7%)	4 (23.5%)		
Father's occupation	Govt. job (n = 6)	0 (0.0%)	3 (50.0%)	3 (50.0%)	χ^2 ^= 27.8	<0.001*
	Private job (n = 22)	0 (0.0%)	15 (68.2%)	7 (31.8%)		
	Business (n = 26)	2 (7.7%)	21 (80.8%)	3 (11.5%)		
	Farmer (n = 63)	15 (23.8%)	44 (69.8%)	4 (6.3%)		
	Unemployed (n = 3)	2 (66.7%)	1 (33.3%)	0 (0.0%)		
Mother's occupation	Govt. job (n = 3)	0 (0.0%)	0 (0.0%)	3 (100.0%)	χ^2 ^= 27.7	<0.001*
	Private job (n = 5)	0 (0.0%)	3 (60.0%)	2 (40.0%)		
	Business (n = 14)	2 (14.3%)	10 (71.4%)	2 (14.3%)		
	Housewife (n = 98)	17 (17.3%)	71 (72.4%)	10 (10.2%)		
Sources of information	Parents (n = 88)	16 (18.2%)	62 (70.4%)	10 (11.4%)	χ^2 ^= 16.9	0.009*
	Siblings (n = 8)	0 (0.0%)	7 (87.5%)	1 (12.5%)		
	Teacher (n = 17)	1 (5.9%)	14 (82.3%)	2 (11.8%)		
	Books (n = 7)	2 (28.6%)	1 (14.3%)	4 (57.1%)		

Observations on personal hygiene practices among the participants

Of the 120 primary school students, 109 (90.8%) had no body odor at the time of observation, and 115 (95.7%) had no ear discharge. Also, 78.3% (n = 94) of the individuals wore clean clothes, 84.2% (n = 101) had a clean face, and 85.8% (n = 103) had clean eyes. Only 76.8% (n = 92) had clean hair, 35.0% (n = 42) had improperly trimmed fingernails, and only 71.7% (n = 86) of participants had clean fingernails. A total of 45 (37.5%) children had nose discharge. Additionally, 46 (38.3%) children had dirty teeth and tongues (Table 8).

**Table 8:** Observation checklist for practices of personal hygiene *p < 0.05 was considered statistically significant for the chi-square test

Sl no	Items	Response	Frequency	Percentage (%)
1	Are the clothes of the child clean?	Yes	94	78.3
2	Are the child's fingernails clean?	Yes	86	71.7
3	Are the child's fingernails trimmed?	Yes	78	65.0
4	Is the child's face clean?	Yes	101	84.2
5	Is the child's eye clean?	Yes	103	85.8
6	Whether the teeth and tongue of the child is clean?	Yes	74	61.2
7	Is the child clean?	Yes	92	76.2
8	No body odor is present in the child	Yes	109	90.8
9	No discharge is present in the ear of child's ear	Yes	115	95.8
10	No discharge is present in the child's nose	Yes	75	62.5

## Discussion

Proper behavior and practice of personal hygiene significantly contribute toward health advancement and illness prevention. Lack of frequent handwashing and microbial contamination of the water at homes and communities increase the danger of transmitting diarrheal and waterborne illnesses. Recently, India has made significant strides in reducing open defecation nationwide, which has a substantial impact on enhancing water, sanitation, and hygiene (WASH) [14]. A recent study among school-going adolescents documented that dietary practices and personal hygiene are significant contributors to undernutrition; therefore, it is strongly advised to address the issue of undernutrition by encouraging healthy behaviors through school health policies that engage parents, teachers, and students [15]. Several studies have demonstrated that school-going children often exhibit poor personal hygiene practices despite possessing a good level of knowledge and a positive attitude [16,17].

The present study included 120 primary school children from seven government schools to assess their knowledge and practices of personal hygiene in a rural area of northeast India. The majority of participants (45.8%, n = 55) belonged to the 9-10 year age group and came from nuclear families (n = 103, 85.8%). More than half (55.0%; n = 66) were females. The present study revealed that parents (n = 88, 73.3%) and teachers (n = 17, 14%) were the primary sources of information regarding personal hygiene among the students. Parents and family have been documented as a major source of information on personal hygiene in other similar studies [17,18].

Most of the children were aware of the importance (n = 109, 90.8%) and measures (n = 117, 97.5%) of maintaining personal hygiene. Also, above 80.0% participants were aware that maintaining proper hygiene is crucial for overall health. The findings of the present study showed that a majority of participants, 58.3% (n = 70), had moderately adequate knowledge of personal hygiene. Despite being aware of the importance of personal hygiene, 70% (n = 84) had moderate practices regarding personal hygiene. Hand hygiene practice among the participants was generally satisfactory, as reported in a similar study [19]. The recent COVID-19 pandemic has substantially increased knowledge and awareness of the importance of hand hygiene among the general population, and handwashing practices have also improved globally [20,21]. Although the majority of the children were aware of dental hygiene (89.2%, n = 107), they mostly practiced regular brushing before breakfast only (97.50%, n = 117).

Brushing teeth after dinner or after consuming sweets, as well as regular dental check-ups, were less frequently practiced. An earlier study conducted among secondary school children also found that brushing twice daily and visiting the dentist are often practiced less frequently by the participants. Dental health education can only be more effective if health programs are designed to directly influence the attitudes of the target population, particularly schoolchildren, who are more easily and persistently instilled with healthy habits [22]. Despite numerous dental health education programs and a variety of oral health maintenance materials being implemented in educational institutions and other settings in recent times, a recent study among rural school children noted that none of the schoolchildren received any interactive oral hygiene practice sessions with their class teachers [23]. Studies have documented a significant association between the prevalence of gingivitis among children and their brushing habits [24]. Research also found that the decayed, missing, filled teeth index (DMFT score) of children who brushed once daily was considerably higher [25]. It is essential to enhance the knowledge, oral hygiene practices, and oral health awareness among school-age children in rural areas. To modify their attitudes, educators and parents must be inspired and educated about dental care. It is advised that parents, teachers, and schoolchildren in remote areas participate in school-based dental health education programs.

In the present study, religion (p < .001) and the occupation of the father (p < .001) were found to be significantly associated with both the knowledge and practice scores of the participants. In certain groups, cultural and religious considerations have a significant impact on both mandatory and optional hand hygiene behaviors [26]. A study conducted by Mangal et al. found that the age of students, parents' literacy, and fathers' income have a significant association with students' hygiene scores [27]. Furthermore, a significant association between practices and sources of information (p < .009) indicates that children practice personal hygiene behaviors more promptly under the supervision of parents and family. A prior study documented that children who were from families affected by the primary characteristics of social inequality were found to have poor hygiene habits; they were also less likely to believe that their family had the most influence on their hygiene practices and were more likely to perceive social rejection as a result [11]. In the present study, the occupation of the mother was also found to be associated with personal hygiene practice (p < 0.001). Previous studies have shown that maternal education has a dynamic influence on the personal hygiene habits of children [28]. In India, the reported personal hygiene behaviors of mothers and caregivers, as well as household sanitation practices, are powerful indicators of child stunting [29].

In the present study, more than 90% of the participants were free from ear discharge and body odor during observation. Eye and face cleanliness was observed in almost 85%. Only 71.7% (n = 86) of participants had clean fingernails, while 35.0% (n = 42) of children had improperly trimmed fingernails. Of the children, 45 (37.5%) had nose discharge. Also, 46 children (38.3%) had dirty teeth and tongues. The observations suggest a significant gap between the knowledge and practice of personal hygiene among children. Laziness, a lack of essential education, poor time management, and inadequate access to water are documented as major barriers to good hygiene practices [1].

To positively influence children's health behaviors, parents and educators should be responsible for teaching children about personal hygiene from a young age. A recent study, which utilized a projector-based visual display to illustrate both good and bad personal hygiene practices, demonstrated a notable improvement among the students [30]. It is important to continue providing primary school students with high-quality health education on personal hygiene as a basic preventive measure against illnesses.

Limitation

The study included only 120 primary school students. Data was collected using a planned interview schedule that only allowed for yes/no responses, which limited the amount of information that could be gleaned from the respondents. Moreover, the investigator had no control over the group. The responses provided by the kids can also be influenced by their classmates. However, to mitigate the situation, students of the same standard were not allowed to sit together. Observation checklists were completed in a separate room, one student at a time. A similar investigation with a larger sample size can be used for generalization. A systematic educational program could be used to determine how effectively STP influences the lifestyle habits of primary school students. Other contexts and age groups could be used to repeat a similar study.

## Conclusions

Most participants were aware of and practiced personal hygiene to a moderate extent. Although personal hygiene practices and knowledge were positively correlated, a significant gap was observed between the reported knowledge and practice of the children and the observed personal hygiene indicators. Enhancing the knowledge of primary school children and providing them with appropriate health education on cleanliness practices is necessary to influence the children's conceptions. Health education initiatives, both within the family and at school, can encourage individuals to adopt hygienic practices, which may even influence their views toward health and well-being for the rest of their lives.

## Appendices

Questionnaire for data collection in English

(Structured interview schedule on personal hygiene among the primary school children)

Instructions: The interviewer will ask questions listed in the schedule, and necessary explanations will be provided. The interviewer will encircle the serial number of the appropriate answer.

The tool was divided into four sections: I, II, III, and IV.

Section I: Sociodemographic characteristics

Section II: Knowledge questions of personal hygiene among primary school children

Section III: Practice questions of personal hygiene among primary school children

Section IV: Observation checklist to identify the practices of personal hygiene among primary school children

Serial No....................

Section I: Sociodemographic characteristics of the participants

1.               Age in years-

a.                7-8

b.                8-9

c.                9-10

d.                >10 years

2.               Gender

a.                Male

b.                Female

3.               Religion

a.                Hindu

b.                Muslim

c.                Christian

d.                Others (Jain, Sikh, Buddhist)

4.               Type of family

a.               Nuclear family

b.               Joint family

c.               Extended family

5.               Standards

a.               3rd standard

b.               4th standard

c.               5th standard

6.               Sources of information

a.                Parents: father/mother

b.                Siblings: brother/sister

c.                Teacher

d.                Television

e.                Books

f.                 Friends

g.               Others specify.............................

7.               Occupation of father

a.                Govt. service

b.                Private service

c.                Business

d.                Farmer

e.                Unemployed

8.                Occupation of mother

a.                Govt. service

b.                Private service

c.                Business

d.                Housewife

Section II

Knowledge questions of personal hygiene among primary school children

Instruction: Each question consists of two options, with positive options that carry a score of one (1) and negative options that carry a score of zero (0).

1. Personal hygiene means maintaining the body's cleanliness

a.           Yes

b.           No

2. Personal hygiene includes taking a bath, washing hands, brushing teeth, using soap, brushing, cutting nails, and caring for gums

a.            Yes

b.            No

3. It is very important to maintain personal hygiene for all

a.            Yes

b.            No

4. Being neat and clean keeps you healthy

a.            Yes

b.            No

5. Taking a bath every day is needed to keep you clean

a.           Yes

b.           No

6. Washing your hands using soap is much better than using water only

a.           Yes

b.           No

7. Maintaining personal hygiene can prevent you from getting diseases

a.           Yes

b.           No

8. Brushing your teeth regularly using toothpaste prevents teeth problems

a.           Yes

b.           No

9. Biting your nail with your teeth is unhealthy

a.            Yes

b.            No

10. Taking too much sugar causes harm to the teeth

a.           Yes

b.           No

11. Hair should be kept clean for the prevention of dandruff

a.            Yes

b.            No

12. You can get the knowledge about personal hygiene from your parents, TV, books, etc.

a.            Yes

b.            No

Section III

Practice questions on personal hygiene among primary school children

1. Do you wash your hands with soap before eating?

a.             Yes

b.             No

2. Do you wash your hands with soap after using the toilet?

a.             Yes

b.             No

3. Do you wash your hands after playing?

a.               Yes

b.               No

4. Do you use soap while washing your hands?

a.             Yes

b.             No

5. Do you use hand sanitizer instead of hand washing in school?

a.               Yes

b.               No

6. Do you brush your teeth before breakfast?

a.               Yes

b.               No

7. Do you brush your teeth after dinner?

a.             Yes

b.             No

8. Do you rinse your teeth after eating sweets?

a.              Yes

b.              No

9. Do you visit the dentist yearly?

a.              Yes

b.               No

10. Do you take a bath regularly?

a.               Yes

b.               No

11. Do you wash your hair daily?

a.             Yes

b.             No

12. Do your parents or a family member help you take a bath?

a.             Yes

b.                 No

Section IV       

                Observation Checklist

1. Are the clothes of the child clean?

a.                Yes

b.                No

2. Are the child's fingernails clean?

a.                    Yes

b.                    No

3. Are the fingernails of the child trimmed?

a.                    Yes

b.                    No

4. Is the face of the child clean?

a.                    Yes

b.                    No

5. Is the eye of the child clean?

a.                    Yes

b.                    No

6. Whether the teeth and tongue of the child are clean?

a.                    Yes

b.                    No

7. Is the child's hair clean?

a.                    Yes

b.                    No

8. No body odor is present in the child

a.                    Yes

b.                    No

9. No discharge is present in the ear of the child

a.                    Yes

b.                    No

10. No discharge is present in the nose of the child

a.                    Yes

b.                    No
